# Natural Polyphenols as SERCA Activators: Role in the Endoplasmic Reticulum Stress-Related Diseases

**DOI:** 10.3390/molecules27165095

**Published:** 2022-08-10

**Authors:** Jana Viskupicova, Petronela Rezbarikova

**Affiliations:** Centre of Experimental Medicine SAS, Institute of Experimental Pharmacology & Toxicology, Slovak Academy of Sciences, 84104 Bratislava, Slovakia

**Keywords:** Ca^2+^ signaling, ellagic acid, ER stress, gingerol, luteolin, resveratrol, SERCA

## Abstract

Sarco/endoplasmic reticulum Ca^2+^-ATPase (SERCA) is a key protein responsible for transporting Ca^2+^ ions from the cytosol into the lumen of the sarco/endoplasmic reticulum (SR/ER), thus maintaining Ca^2+^ homeostasis within cells. Accumulating evidence suggests that impaired SERCA function is associated with disruption of intracellular Ca^2+^ homeostasis and induction of ER stress, leading to different chronic pathological conditions. Therefore, appropriate strategies to control Ca^2+^ homeostasis via modulation of either SERCA pump activity/expression or relevant signaling pathways may represent a useful approach to combat pathological states associated with ER stress. Natural dietary polyphenolic compounds, such as resveratrol, gingerol, ellagic acid, luteolin, or green tea polyphenols, with a number of health-promoting properties, have been described either to increase SERCA activity/expression directly or to affect Ca^2+^ signaling pathways. In this review, potential Ca^2+^-mediated effects of the most studied polyphenols on SERCA pumps or related Ca^2+^ signaling pathways are summarized, and relevant mechanisms of their action on Ca^2+^ regulation with respect to various ER stress-related states are depicted. All data were collected using scientific search tools (i.e., Science Direct, PubMed, Scopus, and Google Scholar).

## 1. Introduction

Calcium (Ca^2+^) is one of the most important regulators of cell survival/death processes. Aberrations in Ca^2+^ homeostasis have been linked to the development of various pathophysiological conditions, such as cardiovascular diseases [1,2], diabetes [3,4], cancer [5], neurodegenerative diseases [6,7], and skeletal muscle pathologies [8]. Intracellular Ca^2+^ concentrations [(Ca^2+^)_i_] must be maintained within very low concentrations (~10–100 nmol/L), which are regulated by a number of Ca^2+^ transport systems, such as pumps and channels [9]. The most important amongst them are the sarco/endoplasmic reticulum (SR/ER) Ca^2+^-ATPase (SERCA) pumps, which transport Ca^2+^ ions from the cytosol into the SR/ER ([(Ca^2+^)_SR/ER_]~100 to 800 μmol/L) in an ATP-dependent manner [10], thus maintaining a steep concentration gradient of Ca^2+^ across the membrane [11]. SERCA pumps are coded by three genes (*ATP2a1*, *ATP2a2*, and *ATP2a3*), which generate several distinct tissue-specific SERCA isoforms (SERCA1–3) through alternative splicing [12]. Currently, more than 14 tissue-specific SERCA mRNA splice variants and their corresponding proteins have been discovered [13]. Impaired Ca^2+^ uptake into cardiac, vascular, and skeletal cells, or decreased SR/ER Ca^2+^ content, is associated with downregulation/reduced activity of the respective SERCA isoforms. These changes further intervene in calcium-release channels (ryanodine receptors (RyRs) and inositol-1,4,5-triphosphate receptors (IP3Rs)) and plasma membrane Ca^2+^-influx channels, such as transient receptor potential canonical channels and calcium-release-activated calcium channels (ORAI), causing disruption of intracellular Ca^2+^ homeostasis [14].

Under certain pathophysiological conditions—including Ca^2+^ homeostasis imbalance, increased secretory load, energy deprivation, impaired redox homeostasis, viral infections, cytotoxicity, inflammation, and mutations [15,16]—unfolded and misfolded proteins may be accumulated in the ER, activating a condition called ER stress. In order to restore ER homeostasis, cells activate secondary adaptive events known as unfolded protein responses, along with related signaling pathways, such as reduced protein loading into the ER, translational attenuation, and correction of protein folding [17]. However, if the stress is persistent, the processes of either apoptotic cell death or autophagy are triggered. The inositol-requiring enzyme 1 with c-Jun NH2-terminal kinase (JNK) and eukaryotic initiation factor 2 alpha with C/EBP homologous protein signaling pathways belong to the main paths of ER stress, which are modulated by different factors involving injured Ca^2+^ homeostasis [18]. ER stress has been recognized to be linked to various disease pathogeneses, including obesity, diabetes, metabolic syndrome, neurodegenerative diseases, and cancer [19,20]. Increasing evidence suggests that a critical role in triggering ER stress is attributed to reduced SERCA2b function, while restoration of SERCA2b leads to the relief of ER stress [21]. Overexpression of SERCA2a by gene therapy has been successfully used in clinical models of heart failure [22].

SERCA function is modulated by various physiological processes and endogenous factors. Primarily, the activity of SERCA pumps is regulated by two small endogenous proteins: phospholamban (PLB) and sarcolipin (SLN), expressed in cardiac and skeletal muscles, respectively. They bind to the regulatory site in SERCA and, thus, reduce the apparent affinity of SERCA1a and SERCA2a for Ca^2+^, in the case of SLN and PLB, respectively [23], although both SLN and PLB may be involved in the modulation of either SERCA isoform [24]. Recently, dwarf open reading frame (DWORF) has been reported as a new endogenous regulator of SERCA [25]. DWORF acts as a direct activator of SERCA, removing PLB as an inhibitor of SERCA and, thus, increasing its turnover rate [26]. ER Ca^2+^ dynamics are also controlled by anti- and pro-apoptotic proteins, such as Bcl-2 family members and protein p53, respectively. Studies demonstrate that Bcl-2 overexpression reduces ER Ca^2+^ levels by inhibiting SERCA2 [27], while the tumor suppressor p53 stimulates SERCA activity, resulting in ER Ca^2+^ overload [28,29]. Additionally, various post-translational modifications and protein–protein interactions of SERCA have been described, leading to enhancement of SERCA activity and Ca^2+^ uptake [30].

Based on current knowledge, SERCA may play a role as a molecular target in ER stress-related diseases [30]. Compounds that are able to positively regulate cellular responses to ER stress may serve as potential drug candidates for various ER stress-related conditions. Restoration of ER Ca^2+^ regulation and management of cytosolic Ca^2+^ levels via SERCA are considered to represent a promising therapeutic strategy in various disease states. There is limited information about the mechanistic behavior of polyphenol-based activators on SERCA pumps or Ca^2+^-related signaling processes. As far as the authors are aware, there is no comprehensive article available regarding polyphenol-mediated SERCA activation. Here, we provide an overview of natural polyphenols able to modulate Ca^2+^ signaling routes and increase the activity/expression of SERCA with respect to intervention in various chronic pathological conditions.

## 2. Intracellular Ca^2+^ Regulation: The Role of SERCA

The endoplasmic reticulum and mitochondria are the main regulators of intracellular Ca^2+^ homeostasis, which is important to maintain a variety of cellular functions. This requires a complex interplay of different Ca^2+^ transporters, along with Ca^2+^-sensing and -buffering proteins, channels, receptors, and their regulators. The low [Ca^2+^]_i_ is maintained due to the action of the plasma membrane Ca^2+^-ATPase (PMCA) and Na^+^/Ca^2+^-exchanger (NCX), which are responsible for the extrusion of Ca^2+^ from the cell. Upon elevated [Ca^2+^]_i_, the SERCA pump is activated to maintain the required [Ca^2+^]_i_ by sequestering Ca^2+^ from the cytosol into the ER [31]. Calcium-permeable channels located on the plasma membrane regulate the entry of Ca^2+^ into the cell. These include voltage-gated calcium channels (VGCCs), which respond to membrane depolarization; receptor-operated channels (ROCs), which are activated by the interaction with ligands; and store-operated calcium channels (SOCs), which are stimulated by the depletion of internal Ca^2+^ stores. The inositol-1,4,5-triphosphate receptor (IP3R) and the ryanodine receptors (RyRs) are the main players in mediating the release of Ca^2+^ from the internal stores. Inositol-1,4,5-triphosphate activates IP3R, triggers the release of Ca^2+^ from stores, and further increases IP3R’s sensitivity to Ca^2+^ [32]. Store-operated Ca^2+^ entry (SOCE) significantly contributes to the dynamics of Ca^2+^ in cells. This Ca^2+^ signaling pathway is activated upon Ca^2+^ store depletion; consequently, the ER Ca^2+^-sensing stromal interaction molecules (STIM1 and STIM2) oligomerize, move to the ER membrane, and bind to the calcium-release-activated calcium (CRAC) channel proteins ORAI1, ORAI2, and ORAI3, located in the plasma membrane, allowing pore opening for Ca^2+^ to enter the cell [33]. Some members of TRPC (transient receptor potential canonical) channels may also contribute to a store-operated current [34]. Inter-organelle communication between the ER and mitochondria is mediated via mitochondria-associated ER membranes (MAMs) in a highly organized manner. There is a diverse group of several critical proteins involved in ER–mitochondria tethering—especially mitofusin 2, responsible for bridge formation between mitochondria and the ER; the interaction of IP3R with voltage-dependent anion channel 1; the mitochondrial Ca^2+^ uniporter (MCU) complex, involved in coupling between cytosolic/MAM Ca^2+^ signaling and the activation of key dehydrogenase enzymes for energy generation; and others, as reviewed in [35].

SERCA pumps, together with PMCAs and NCXs, are among the most important regulators responsible for restoring low resting [Ca^2+^]_i_. In certain tissues, SERCA sequesters more than 70% of the cytosolic Ca^2+^ [23]; therefore, it plays a crucial role in maintaining intracellular Ca^2+^ homeostasis. The primary structures of SERCAs are highly conserved, and individual SERCA isoforms possess a high percentage of sequence homology. Functional differences between SERCA isoforms consist of their affinity for Ca^2+^ (2b > 2a = 1 > 2c > 3) and their Ca^2+^ transport turnover rates [13]. Individual SERCA isoforms are tissue-specific, and the impairment in their regulation has been associated with various disease states, as shown in Table 1.

Since the decreased [Ca^2+^]_ER_, rather than the increased [Ca^2+^]_i_, triggers apoptosis, it has been suggested that maintaining ER Ca^2+^ homeostasis via ER-localized pumps and channels represents a primary stimulus in triggering mechanisms leading to aberrations of intracellular Ca^2+^ homeostasis, as well as to the onset of ER-stress-related diseases [39]. These changes are very much dependent on the cell type and the disease model studied. SERCAs, together with STIM1 and ORAI1, contribute to capacitative Ca^2+^ entry, which is responsible for refilling of the SR/ER stores, with Ca^2+^ entering cells via activated SOCs [44]. Moreover, the involvement of TRPC1 was reported to contribute to the SOCE pathway in skeletal muscle [45]. The SERCA1 isoform is regulated by STIM1 through direct binding to SERCA1 via the C-terminal part; thus, STIM1 is involved in maintaining SERCA1 activity [46]. In human platelets, SERCA2b and SERCA3 are responsible for the direct regulation of SOCE via the hTRPC1 channel [47], demonstrating strong interplay between SOCE-related proteins and SERCAs. Under conditions of ER stress, the stimulatory interaction between STIM1 and SERCAs was found to be impaired [48]. Defective Ca^2+^ loading into the SR/ER caused by SERCA dysfunction may be partially compensated by other regulatory mechanisms, such as extrusion of Ca^2+^ via PMCAs and NCXs, uptake of Ca^2+^ into the mitochondria or Golgi apparatus, or upregulation of TRPC1 [36]. However, if cellular adaptive mechanisms directed towards balancing Ca^2+^ homeostasis fail, multiple ER stress-related pathologies can be induced.

## 3. Polyphenol–SERCA Interactions

Polyphenols comprise a diverse group of secondary plant metabolites, which are widely distributed in nature. They are found predominantly in various fruits, vegetables, cereals, green tea, and red wine. The major classes of dietary polyphenols comprise flavonoids, stilbenes, lignans, and phenolic acids [49]. They are generally considered to be safe, with a lower degree of bioavailability in humans. These phytochemicals are known for a wide range of health-promoting properties; mainly listed are their antioxidant, anti-inflammatory, anti-carcinogenic, and neuroprotective activities. A polyphenol-rich diet has been associated with decreased risks of developing cancer, cardiovascular diseases, cerebrovascular diseases, diabetes, osteoporosis, and neurodegenerative diseases [50,51]. Moreover, polyphenols have been reported to act as modulators of cellular signaling and regulatory factors of gene transcription, thus intervening in various intracellular processes [52]. However, information on their molecular interactions with protein targets is limited. This is an important step to address in order to understand the precise mechanism of their action. In silico evidence suggests that various polyphenols may interact with ATP-binding cassette transport systems (ABC transporters) [53]. Direct binding of these phytochemicals to membrane transporters, including P-type ATPases [52], has been described as an effective health-promoting means of polyphenol-mediated protective action [54].

SERCA belongs to the most studied membrane transporters. The catalytic transport cycle of SERCA has been described by the E1–E2 scheme, starting with the high-affinity Ca^2+^-binding site (E1), followed by phosphorylation of the Ca^2+^-ATPase by ATP (E1~P) and conversion into E2P, ending up with enzyme dephosphorylation (E2) [55]. The inhibitory actions of polyphenols on different isoforms of SERCA have been extensively examined, and their mechanisms of action have been proposed [56,57,58,59,60,61]. Thapsigargin, the most potent natural SERCA inhibitor, acts through noncompetitive inhibition of SERCA by blocking SERCA in the E2 conformation at sub-nanomolar concentrations [61]. Interestingly, thapsigargin selectively inhibits all known SERCA isoforms, but fails to show a similar inhibitory effect on PMCA—a highly homologous protein to SERCA [62]. The inhibitory mechanisms of several polyphenols—such as curcumin [60,63], epigallocatechin-3-gallate (EGCG) [56,59], or flavonol quercetin [58,64,65]—in the low micromolar concentration range have been well described. Curcumin, a polyphenol of turmeric, was reported to inhibit SERCA activity by stabilizing the E1 conformational state of SERCA and preventing ATP binding [60]. EGCG has a preferential interaction with the E2 conformation, affecting the enzyme at the catalytic site [56]. Quercetin and structurally similar flavonoids have been described to stabilize SERCA in the E1 conformation, reducing ATP binding [58]. We previously found that both quercetin and rutin derivatives reduced SERCA1 activity in a concentration-dependent manner, and caused structural and conformational changes in the SERCA1 protein [66,67,68]. Recently, we showed that phenolic compounds from *Morus nigra* modulated the viability and apoptosis of INS-1E pancreatic beta cells via SERCA2 activity [69]. Selective SERCA inhibition by natural products has been recognized as a useful tool to trigger targeted ER stress and apoptosis in cancer cells. Interestingly, the inhibition of SERCA pumps was recently recognized as an efficient anti-aging strategy that supports longevity, suggesting that Ca^2+^ signaling may have an impact on aging [70].

## 4. Pharmacological Activation of SERCA by Polyphenols

**Figure 1:**
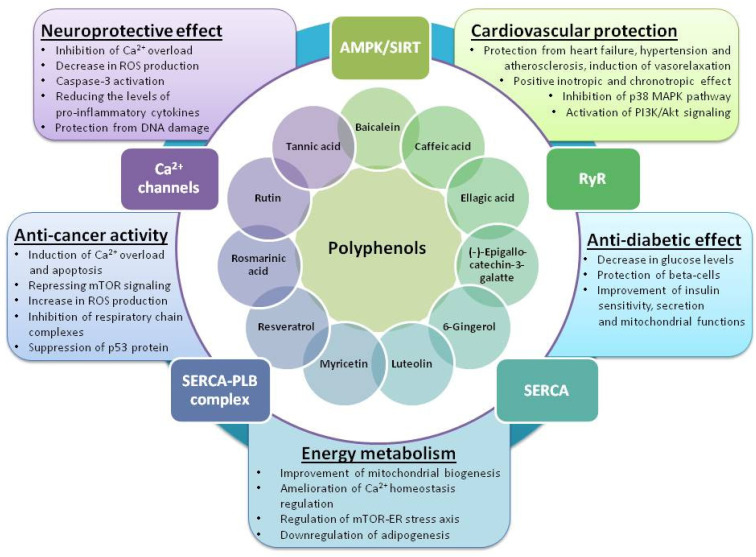
Ca^2+^-dependent protective effects of polyphenols linked to ER stress-related diseases.

Pharmacological activation of SERCA can reduce ER stress, and may therefore represent a promising therapeutic approach for the treatment of diabetes, metabolic disorders, cardiovascular diseases (especially heart failure), and neuropathological conditions; alternatively, induction of ER stress by polyphenols may contribute to cancer treatment, as illustrated in Figure 1.

Natural polyphenols are able to specifically modulate Ca^2+^ homeostasis and Ca^2+^ signaling pathways via SERCA. Polyphenols can affect SERCA by direct binding [71], followed by subsequent changes in its structure and activity. In addition, indirect mechanisms may also lead to alterations in SERCA expression and/or activity. Polyphenol-mediated conformational alterations in either the ATP-binding or Ca^2+^-binding sites of SERCA are crucial for their protective effects in vivo [72].

To date, most studies on SERCA activation have been conducted regarding the quinoline derivative CDN1163 [73,74]. This small molecular allosteric SERCA activator balances disrupted Ca^2+^ homeostasis and attenuates diseases associated with ER stress, such as diabetes, metabolic disorders, neurodegenerative problems, or muscular dystrophy [75,76,77]. Other drug-like SERCA activators, including istaroxime and pyridone derivatives, have been reported to possess stimulatory effects on the cardiac SERCA2a isoform, making them applicable in heart failure treatment [78]. However, there is little information available on the activation of SERCA by natural compounds. Resveratrol, gingerol, ellagic acid, and luteolin belong to the most listed SERCA-targeting compounds in the literature. Table 2 summarizes up-to-date information regarding the effects of polyphenols related to SERCA activation. These compounds exhibit diverse mechanisms of action on the Ca^2+^ regulatory machinery, from direct interaction with SERCA, through indirect effects via inhibition of SERCA–PLB complex formation, to complex intervention in Ca^2+^ signaling pathways, thus contributing to various health effects.

**Figure 2:**
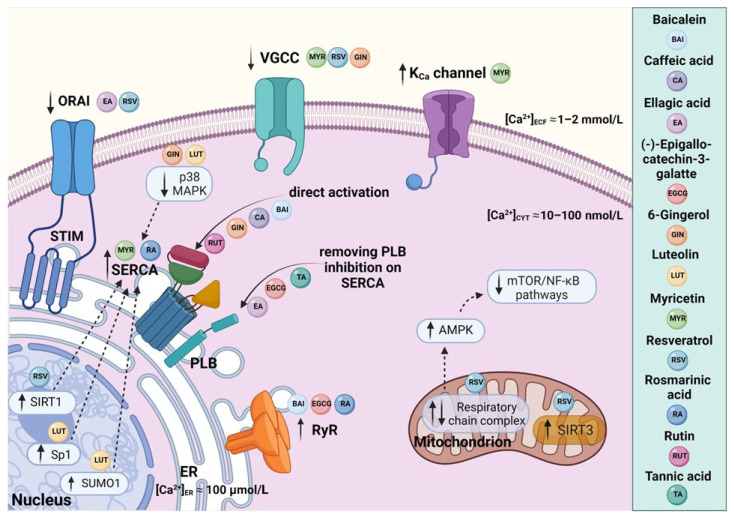
A schematic representation of polyphenol-mediated effects on SERCA and related intracellular Ca^2+^ signaling pathways: Dietary polyphenols affect Ca^2+^ dynamics by targeting Ca^2+^ transporters and channels as well as downstream processes. Baicalein, rutin, caffeic acid, and gingerol seem to stimulate SERCA directly. On the other hand, ellagic acid, (-)-epigallocatechin-3-gallate, and tannins were described as indirect SERCA activators acting by relieving the inhibition of SERCA by PLB. Resveratrol has been shown to interact with several Ca^2+^-handling proteins, and to modulate Ca^2+^ homeostasis through intervention in Ca^2+^ signaling pathways. In particular, the activation of deacetylase SIRT1 has been reported as a central mechanism of resveratrol action responsible for upregulation of SERCA. The release of Ca^2+^ from the ER via RyRs was shown to be facilitated by baicalein, (-)-epigallocatechin-3-gallate, and rosmarinic acid. Luteolin, myricetin, and rosmarinic acid increase the overexpression of SERCA. The regulatory effects of myricetin, resveratrol, gingerol, and ellagic acid were described in relation to Ca^2+^-dependent channels, such as VGCCs, ORAI–STIM, and the K_Ca_ channel. Figure created with BioRender.com.

A schematic representation of the major mechanisms of polyphenols’ action with respect to intracellular Ca^2+^ signaling is depicted in Figure 2.

Detailed information on individual polyphenol-mediated Ca^2+^-dependent mechanisms is provided in the following sections.

### 4.1. Resveratrol

Resveratrol (3,4′,5,-trihydroxystilbene, RSV), a polyphenolic stilbene, is a secondary plant metabolite found mainly in grapes and red wines. It contains two phenyl rings joined by an ethylene bridge. Studies have reported substantial biological health effects of this compound. These include antioxidant, anticancer, anti-inflammatory, anti-carcinogenic, antidiabetic, cardioprotective, immunomodulatory, vasorelaxant, phytoestrogenic, and neuroprotective activities [102,103,104,105]. Moreover, clinical data regarding the pharmacological action of RSV show that this phytomolecule beneficially influences neurological disorders, cardiovascular diseases, diabetes, obesity, and cancer [102,104]. Plasma levels of RSV were found to be below the micromolar range [106]. Owing to the lipophilic character of the RSV molecule, it is expected to have affinity for membranes; thus, its cellular levels might be higher [107].

RSV has been reported to act as a multitarget signaling molecule. It modulates cellular Ca^2+^ homeostasis by affecting intracellular Ca^2+^ pumps, Ca^2+^ channels, and/or Ca^2+^ signaling pathways. RSV may act as a direct ligand for Ca^2+^-sensing transmembrane proteins, such as L-type and T-type VGCCs. On the other hand, Ca^2+^-handling proteins such as SERCA and PMCA constitute indirect targets of RSV [105]. RSV causes an increase in [Ca^2+^]_i_ by depletion of intracellular Ca^2+^ stores, mainly due to the activation of capacitative Ca^2+^ entry and the involvement of other Ca^2+^-permeable channels [108]. RSV was found to improve overall Ca^2+^ homeostasis in various conditions of cellular dysfunction. Direct inhibition of L-type VGCCs and indirect inhibition of Ca^2+^-activated potassium channels by RSV may lead to vasorelaxation, which may be beneficial in preventing cardiovascular diseases such as hypertension and atherosclerosis [105].

It seems that RSV acts on multimodal signaling cascades. One of the key mechanisms through which RSV manifests its protective effects is the activation of deacetylases. It is a potent activator of the NAD+-dependent histone deacetylase SIRT1 [109], which is associated with the regulation of various cellular functions. Therefore, RSV-mediated modulation of SIRT expression/activity may represent an effective therapeutic strategy to combat various chronic metabolic and inflammatory diseases [96,110]. The antidiabetic effect of RSV has been associated with its ability to decrease blood glucose levels, preserve beta-cell function, and improve insulin sensitivity and secretion [111]. A dramatic reduction in SIRT1 levels has been observed in diabetic patients [112]. Sulaiman et al. [97] showed that RSV prevented the diabetes-induced decline in SERCA2a, improved cardiac function, and enhanced SERCA2 promoter activity in cardiomyocytes under hyperglycemia. Based on these findings, it seems that RSV modulates SERCA2a expression and improves cardiac function via SIRT activation [97].

RSV is able to influence various processes on the level of the mitochondria and energy metabolism [102]. RSV treatment has been reported to enhance the expression of the mitochondrial deacetylase SIRT3 [113], which plays a role in energy metabolism, as well as in the regulation of mitochondrial respiration rate and ATP production. In particular, improvement of oxidative phosphorylation in diabetic cardiomyopathy by RSV via SIRT3 has been reported [114]. From the available data, it seems that the activity of SIRT is tightly interlinked with AMP-activated protein kinase (AMPK). The activation of the AMPK/SIRT pathway by RSV leads to inhibition of the mTOR and NF-κB pathways [107]. The suggested mechanism by which RSV may activate AMPK is based on the ability of RSV to directly bind to the mitochondrial respiratory chain complexes, such as NADH dehydrogenase (complex I) [115] or F0F1-ATPase/ATP synthase (complex V) [107,116]. It has been suggested that the effect of RSV on respiratory chain complexes is concentration-dependent [117]. While low RSV concentrations (1–5 μmol/L) stimulated respiratory chain complex I activity in a human hepatoblastoma cell line, high concentrations of RSV (>50 μmol/L) resulted in inhibition of complex I activity [118]. The mechanisms of SIRT1 and AMPK activation, as well as deacetylation of peroxisome proliferator-activated receptor gamma coactivator 1-alpha by RSV, have been reported to be connected with the improvement of mitochondrial biogenesis and energy metabolism [119]. Both the AMPK and mTOR signaling pathways are tightly connected to Ca^2+^ regulation. An increase in [Ca^2+^]_i_ leads to mTOR inhibition, inducing the process of autophagy [70]. RSV was reported to induce autophagic flux via both mTOR-dependent and -independent mechanisms involving mitochondrial Ca^2+^ signaling pathways, and depending on the presence of IP3R and cytosolic Ca^2+^, suggesting that IP3R channels and intracellular Ca^2+^ signals are essential to trigger autophagy in response to various stimuli [120].

Recent studies have shown that RSV can be an effective agent in fighting cardiovascular diseases, such as cardiac hypertrophy, contractile dysfunction, atherosclerosis, and related vascular complications, by targeting the calcium-regulatory pathway [98,121,122]. A study by Dong et al. [98] showed that RSV significantly ameliorated cardiac hypertrophy and contractile dysfunction induced by pressure overload via prevention of the impairment of Ca^2+^-handling proteins, including SERCA2, RyR2, NCX1, and Ca^2+^/calmodulin-dependent protein kinase II (CaMKII) [98]. The mechanism of RSV-mediated protective action on high-glucose-induced apoptosis of vascular endothelial cells has been reported to rely on the regulation of SOCE. Since high-glucose treatment is associated with upregulation of SOCE-related proteins—including TRPC1, ORAI1, and STIM1 [123]—RSV may manifest its anti-apoptotic effect through restoration of SOCs [121]. RSV was also found to ameliorate the apoptosis of synoviocytes in an adjuvant arthritis rat model by inhibiting ORAI1 expression, thus affecting ORAI1–STIM1 complex formation and Ca^2+^ entry [122].

RSV is also known to trigger apoptotic cell death in cancer cells at higher concentrations (>50 μmol/L) [117]. The suggested mechanisms of the pro-apoptotic effect of RSV include changes in Ca^2+^ regulation, increased ROS levels, disruption of the mitochondrial membrane potential, inhibition of respiratory chain complexes, and caspase activation [117,124]. Another proposed mechanism of RSV-mediated anticancer action is based on the inhibition of ATP synthase; consequently, the required amount of ATP for malign cells is reduced [125]. As a result of the decreased mitochondrial content of ATP, the activity of SERCA pumps was lowered, causing the accumulation of Ca^2+^ within mitochondria-associated ER membranes, and triggering mitochondrial Ca^2+^ overload and apoptosis [124,125,126]. Enhanced sequestration of Ca^2+^ into the mitochondria selectively activated Letm1-dependent Ca^2+^ uptake via MCU in cancer cells. A similar effect was observed in a structural analog of RSV—piceatannol [124].

Recently, Izquierdo-Torres et al. [127,128] contributed to better understanding of the molecular mechanisms of the chemopreventive action of RSV. The authors showed that RSV upregulated *ATP2A3* gene expression in breast cancer cells [127,128]. Changes in the expression of SERCA genes led to the disruption of Ca^2+^ homeostasis and induction of apoptosis, suggesting a critical role of Ca^2+^-dependent pathways in RSV-induced cell death. Activation of *ATP2A3* gene expression was correlated with (i) reduced histone deacetylase 2 nuclear expression, (ii) reduced global histone deacetylase activity, and (iii) enhanced global histone acetyltransferase activity [127]. Interestingly, the anti-apoptotic protein Bcl-2 was overexpressed after RSV treatment. These studies point to the importance of *ATP2A3* gene expression in cancer therapy [127,128]. Additionally, RSV-mediated cancer prevention has also been closely associated with the suppression of the pro-apoptotic p53 protein [129]. A methoxylated structural analogue of resveratrol, (Z)3,4,5,4′-trans-tetramethoxystilbene, is another promising drug with anticancer activity. This stilbenoid was found to directly bind to SERCA and raise the intracellular Ca^2+^ level independently of caspase activation in lung cancer cells. This RSV derivative suppressed the AMPK/mTOR pathway and activated JNK—the cross-linker of ER stress [130].

### 4.2. 6-Gingerol

6-Gingerol [5-hydroxy-1-(4-hydroxy3-methoxyphenyl)decan-3-one; (GIN)] is a major biologically active constituent of ginger. This polyketide is found in the Zingiberaceae family and in the grains of paradise as well as an African ginger species. It has been described to exhibit various effects—most importantly anticancer, anti-inflammatory, and antioxidant properties [131]. The anti-inflammatory action of GIN is mainly attributed to its ability to target the NF-κB pathway [132,133,134]. GIN has received considerable attention as a potential therapeutic agent for the prevention and/or treatment of various—especially inflammation-associated—disorders, including cardiovascular diseases, diabetes, metabolic syndrome, and neurodegenerative diseases [135,136].

Changes in SERCA2a activity/expression and decreased Ca^2+^ uptake into the SR lead to aberrant Ca^2+^ handling in failing hearts, including abnormalities in systolic and diastolic functions [13]. Cardiotonic agents able to activate the SERCA2 isoform could effectively target heart failure. GIN was first described as a potent cardiotonic agent with the ability to activate the SERCA enzyme in 1987 [88]. Moreover, inotropic and chronotropic effects of GIN were also previously reported [137]. GIN (3–30 μmol/L) activated both the SERCA1 and SERCA2 isoforms, with effective concentrations (EC_50_) of 4.0 and 4.3 μmol/L for skeletal and cardiac SR, respectively. These results point to direct stimulatory action of GIN on the SR’s Ca^2+^ uptake, which may be related to the direct activation of SERCA at the regulatory ATP-binding site [88]. Namekata et al. [82] investigated the effect of GIN-mediated SERCA activation on diastolic dysfunction in streptozotocin-induced diabetic mice. Both the rate of relaxation and the rate of Ca^2+^ transient decay were accelerated. The acceleration of relaxation by GIN was completely inhibited by a specific inhibitor of SR Ca^2+^ uptake, cyclopiazonic acid, implying direct action on SERCA [82]. Activation of the SERCA2 pump by GIN was studied in relation to possible regulation by PLB [84]. In cardiac muscle, the SERCA2a isoform is regulated by the endogenous transmembrane protein PLB, which inhibits Ca^2+^ transport at low diastolic [Ca^2+^]. The highest SERCA activity is gained at high systolic [Ca^2+^] or when PLB is phosphorylated [138]. A significant increase in V_max(Ca)_ caused by GIN was observed in both phosphorylated and unphosphorylated SR vesicles, while K_m(Ca)_ was only increased in phosphorylated microsomes. Increased maximal rates by GIN may be attributed to the competition against the inhibition of ATP-accelerated steps by PLB during the transport cycle. Direct activation of the SERCA pump by GIN was outlined, which may play a role in GIN-mediated cardiac contractile responses [84]. Recently, GIN was found to improve pressure-overload-induced cardiac remodeling, which was associated with inhibition of the p38 mitogen-activated protein kinase (MAPK) pathway [139]. Since activated p38 MAPK is involved in pro-apoptotic events via p53 activation, leading to impairment of myocyte function [140], the abovementioned abolishment may be of pharmacological importance in cardioprotection.

Ginger constituents, including gingerols, have been reported for their possible preventive and curative effects against neurodegenerative diseases via their interaction with various molecular targets. GIN-mediated Ca^2+^ channel blockade and cholinesterase inhibition were suggested to contribute to protection against Alzheimer’s disease [141]. Possible involvement of SERCA in the neuroprotective action of GIN under conditions of ER stress was examined [89]. GIN was able to stimulate SERCA activity in SR microsomes (maximal activation at 30 μmol/L) and restore its function in the presence of SERCA inhibitors. However, GIN failed to promote Ca^2+^ uptake and protect neuronal cells from thapsigargin- and cyclopiazonicacid-induced ER stress and subsequent cell death. On the other hand, GIN (50 μmol/L) itself evoked cell death in a neuronal cell line, which was not accompanied by generation of ROS or caspase activity [89]. However, only a single, rather high concentration of GIN was tested which, in the light of hormesis, may have been responsible for the deleterious action of GIN.

GIN was also shown to have impact on Ca^2+^-dependent channels. Cai et al. [142] investigated the effect of GIN on colonic motility and the involvement of the L-type Ca^2+^ channel in rats. They found that this compound mediated concentration-dependent inhibition of spontaneous contraction of colonic longitudinal myocytes by preventing the influx of Ca^2+^ through L-type Ca^2+^ channels [142].

Moreover, ginger extract (with abundant content of 6-gingerol and 6-shogaol) was recently shown to trigger the AMPK/SIRT1 pathway, which was associated with the control of ER stress and the mTOR pathway. Ginger extract effectively influenced energy metabolism via AMPK/SIRT1 with the involvement of Ca^2+^ homeostasis regulation, and may thus be useful in the management of obesity and associated metabolic complications [143].

Other gingerols (e.g., [8]- and [10]-gingerol) and their synthetic analogues have been shown to increase the SERCA activity of skeletal muscle SR in a concentration-dependent manner [144]. Another structural analog related to gingerol, 1-(3,4-dimethoxyphenyl)-3-dodecanon, increased both V_max(Ca)_ and K_m(Ca)_, and decreased the Hill coefficient, as shown previously [145].

### 4.3. Ellagic Acid

Ellagic acid (2,3,7,8-tetrahydroxy-chromeno [5,4,3-cde]chromene-5,10-dione; (EA)) is a dimeric derivative of gallic acid, which is widely distributed in many fruits (mainly berries) and nuts. It structurally consists of both a lipophilic moiety (biphenyl) and a hydrophilic part (hydroxyl groups and two lactone rings), contributing to the amphiphilic properties of EA. EA has been extensively studied due to its potent antiproliferative effects in some types of cancer, together with its antioxidant and anti-inflammatory properties. The increased intake of EA is associated with improvement of obesity and related metabolic complications [146].

Moreover, potent stimulation of SERCA2 activity by EA has been reported. The mechanism by which EA increased SERCA2 activity was predominantly associated with the displacement of PLB inhibition from SERCA [82,83]. Removal of the inhibitory interaction between SERCA2a and PLB was reported to favor the conformational transition E2 to E1, accelerating Ca^2+^ cycling [78]. EA (EC_50_ ≈ 3 μmol/L) was found to stimulate Ca^2+^ uptake and ATP hydrolysis at sub-micromolar Ca^2+^ concentrations in the cardiac SR. SERCA2 activation was associated with the ability of EA to disrupt the interaction between PLB and SERCA2, thereby increasing enzyme turnover [83]. It was previously indicated that structural and functional coupling of SERCA was affected by PLB binding to the nucleotide domain of SERCA. Phosphorylation of PLB contributes to the conformational changes and functional interactions between the nucleotide active sites within oligomeric complexes of individual SERCA molecules. These interactions are required for efficient Ca^2+^ transport and maximal SERCA activity [147]. Similar findings in kinetic measurements were reported by Antipenko et al. [84], who showed that EA increased the maximal rate of microsomal Ca^2+^ uptake and evoked an insignificant decrease in K_m(Ca)_ in purified cardiac SR vesicles [84]. These results are in accordance with those observed by Berrebi-Bertrand et al. [145] in crude cardiac SR vesicles. EA caused an increase in the V_max(Ca)_ of Ca^2+^ uptake and a slight decrease in K_m(Ca)_ in crude cardiac microsomes, associated with the removal of the inhibitory effect of PLB (indirect action of EA on SERCA). However, increased Ca^2+^ uptake into the SR was not accompanied by a parallel increase in SERCA activity in the skeletal SR [145], which might be attributed to the lower extent of sample purification and unspecific interactions. Moreover, the method of Pi liberation—which measures the total SERCA activity, and is therefore considered less specific than NADH-coupled enzyme assay—was used to assess SERCA activity.

SERCA activity and expression were found to be dramatically reduced in diabetes and related complications, such as cardiomyopathy. Decreased expression levels of SERCA2b and SERCA3 have been reported in the beta cells of diabetic patients, resulting in injured insulin secretion [148]. Namekata et al. [82] investigated the effect of EA on SERCA activation in isolated myocardia from streptozotocin-induced diabetic mice. EA accelerated the rate of relaxation and the rate of Ca^2+^ transient decay in this model. The results point to the significance of the protection of SERCA function by specific SERCA activators in alleviation of the pathogenesis of diabetes and its complications [82].

Recently, the anti-inflammatory action of EA was associated with positive regulation of intracellular Ca^2+^ homeostasis by targeting the SOCE pathway [149]. The authors found that EA stimulated IP3R and inhibited SOCE-mediated Ca^2+^ influx in Jurkat T cells via disruption of ORAI–STIM complex formation. The decrease in Ca^2+^ influx led to the reduction in the nuclear factor of activated T-cell translocation and suppression of cytokine expression, suggesting that the described mechanism may be involved in the anti-inflammatory behavior of EA. These results indicate that EA directly targets SOCE-mediated Ca^2+^ influx [149]. Similarly, gallic acid—a monomeric unit of EA—induced [Ca^2+^]_i_ increase via interaction with SOCE, and activated the mitochondrial apoptotic pathway in human glioblastoma cells, pointing to its possible antitumor potential [150].

### 4.4. Luteolin

Luteolin (3′,4′,5,7-tetrahydroxyflavone; LUT), a natural flavone found in many plant species, is the most common flavonoid. Luteolin-rich dietary sources include carrots, radicchio, peppers, celery, oregano, thyme, peppermint, rosemary, and juniper berries. The results of preclinical studies indicate that LUT possesses a wide range of pharmacological properties, such as antioxidant, anti-inflammatory, antimicrobial, and antitumor health effects [151].

Cardioprotective effects of LUT have also been reported. Increased intake of LUT was associated with reduced risk of acute myocardial infarction [152], which may be partially associated with alterations in the function of the SERCA pump caused by PLB inhibition [138]. According to the study of Hu et al. [91], LUT significantly improved cardiac dysfunction by restoring contractility and Ca^2+^ transients, increasing SERCA2a’s expression, activity, and stability in failing cardiomyocytes. LUT also upregulated the expression of the small ubiquitin-related modifier SUMO1 [91]. It has been recognized that SUMOylation belongs within the key post-translational modifications required for regulating SERCA2a function, and that SUMOylation of SERCA2a was markedly lowered in patients with heart failure [153]. LUT was able to increase the phosphorylation of PLB, as well as SUMOylation of SERCA2a [91]. It has been proposed that LUT contributes to the enhancement of SERCA2a’s stability via SUMOylation at Lys585 in a murine model of SERCA [90]. Under conditions of myocardial ischemia–reperfusion injury in mice, LUT was reported to exert cardioprotective effects by modulating SERCA2a’s function and transcriptional activity via the upregulation of the transcription factor Sp1, thus improving ischemia–reperfusion injury of the myocardium. Sp1 overexpression increased the expression of SERCA2a at the transcriptional level [92]. Another mechanism by which LUT increases SERCA2a activity and reduces Ca^2+^ overload was suggested to be via suppression of the activation of the p38 MAPK pathway, as shown after ischemia–reperfusion injury in rat hearts and cardiomyocytes [93]. Inhibition of the p38 MAPK pathway by LUT was connected with increased PLB phosphorylation, thereby enhancing SERCA2a activity and restoring intracellular Ca^2+^ homeostasis [154]. Moreover, LUT was shown to relieve ischemia–reperfusion injury by suppression of apoptosis via activation of the phosphatidylinositol 3 kinase/protein kinase B (PI3K/Akt) signaling pathway and increasing the phosphorylation of Akt, resulting in improvement in SERCA2a activity, albeit without affecting the SERCA2a expression levels in cardiomyocytes [94]. Positive regulation of SERCA2a by LUT contributes to increased Ca^2+^ uptake and restoration of Ca^2+^ homeostasis, leading to improved myocyte contraction in the condition of heart failure [91].

### 4.5. Other Polyphenols

Flavonoids, green tea polyphenols, tannins, and phenolic acids belong to the other phenolic compounds studied in relation to their SERCA-activating properties.

Flavonoids belong to the most studied group of natural compounds for their numerous health-promoting effects. Moreover, these compounds affect many signaling pathways due to their potential to bind to the ATP-binding site of many proteins, including ATPases [155]. The affinity of flavonoids for transmembrane protein targets is also dependent on the degree of their lipophilicity, where the incorporation of flavonoids into lipid bilayers plays a role [156]. Our research group previously found that higher concentrations of rutin (RUT) stimulated SERCA1 activity in rabbit skeletal muscle. According to kinetic analysis, in the presence of RUT (250 and 350 μmol/L), the maximal rate of enzyme catalysis increased and the affinity of SERCA1 for ATP decreased. RUT interacted with the cytosolic ATP-binding region of SERCA1, as confirmed by decreased FITC fluorescence as well as by in silico molecular docking [100].

Another member of flavonoid class, myricetin (3,3′,4′,5,5′,7-hexahydroxyflavone; MYR), is mainly known for its potent antioxidant, anti-inflammatory, antiviral, antitumor, and antidiabetic properties [157]. In relation to type 2 diabetes, MYR was found to protect beta cells from high-glucose-induced apoptosis by inhibiting ER stress. The mechanism of its action involved the inactivation of cyclin-dependent kinase 5 and consequent upregulation of SERCA2b, which was associated with increased transcription and expression levels of transcription factor pancreatic duodenal homeobox 1 (PDX1), playing a crucial role in beta-cell survival. Moreover, MYR prevented thapsigargin-induced ER stress in beta cells [95], suggesting potential therapeutic application of MYR in the treatment of diabetes. In another study, MYR was reported to protect rat neurons by inhibiting glutamate-induced intracellular Ca^2+^ overload, ROS production, and caspase-3 activation, pointing to promising anti-neurodegenerative properties of MYR [158]. Moreover, MYR treatment enhanced Ca^2+^-dependent potassium channel currents in hypothalamic neurons [159]. Another study revealed that MYR may provide cardioprotective effects by inhibiting VGCCs [160]. Fusi et al. [161] showed that MYR facilitated vasoconstriction of vascular smooth muscle by activating L-type Ca^2+^ channels [161]. Studies of MYR-mediated stimulation of Ca^2+^ channel currents revealed that MYR behaved as a Ca^2+^ channel agonist with high affinity for the channel in the inactivated state [162]. It has been suggested that MYR affects Ca^2+^ currents by activating T- and L-type Ca^2+^ channels through the involvement of CaMKII [163]. Taken together, the above-described complex interplay of MYR’s action at various levels within the Ca^2+^ signaling cascade may ameliorate disease-driven Ca^2+^ dysfunction.

Polyphenols have been reported to play an important role in suppressing the development of heart failure by improving Ca^2+^ regulation in myocytes [164]. Baicalein (5,6,7-trihydroxyflavone; BAI) is a type of flavonoid originally isolated from the roots of *Scutellaria baicalensis* and *Scutellaria lateriflora*, and possesses many pharmacological effects, such as antiviral, antioxidant, anti-inflammatory, anticancer, and cardioprotective activities [165,166]. Zhao et al. [79] studied the effects of BAI on elevated pressure in heart failure and possible involvement of Ca^2+^-handling proteins in Ca^2+^ dysregulation. They showed that BAI prevented pressure overload in vivo, partially due to modulation of SERCA activity, as well as upregulation of SERCA2 and RyR2 [79]. Moreover, BAI affected downregulation of phosphorylation of CaMKII and expression of NCX. These findings point to multimodal Ca^2+^-regulatory effects of BAI on myocardial remodeling. The protective action of BAI against myocardial ischemic injury was also reported by another research group; however, the underlying protective mechanisms of BAI were mainly attributed to its anti-inflammatory and antioxidant properties [80].

Flavonoids have also been reported to affect Ca^2+^-regulatory processes indirectly, via modulation of sirtuins [167]. SIRT6 plays a role in Ca^2+^ signaling via the synthesis of Ca^2+^-mobilizing second messengers, affecting Ca^2+^-dependent transcription factors and responses, as well as the expression of pro-inflammatory cytokines. Therefore, SIRT6 modulation may be an effective approach in the treatment of cancer by targeting inflammation, angiogenesis, and metastasis [168]; diabetes via improvement of insulin secretion [169]; and neurodegenerative disorders via neuroprotection from DNA damage [170]. According to a recent study, the polyphenols isoquercetin, luteolin, and cyanidin activate SIRT6, while vitexin, catechin, scutellarin, and fucoidan act as SIRT6 inhibitors. Quercetin displayed both activating and inhibitory effects on SIRT6, depending on the concentration used [171]. Moreover, upregulation of SIRT1 and SIRT2 expression levels in vivo by quercetin was also observed [172].

Green tea is a rich source of polyphenols, of which EGCG is the best-studied tea component, with multiple beneficial actions in different human pathological states. EGCG was described as affecting second messenger Ca^2+^ signals and raising cytosolic Ca^2+^ levels, thereby modulating intracellular Ca^2+^ signaling pathways. As a result, EGCG (<2 μmol/L) increased the sensitivity of RyR1 in response to inward Ca^2+^ current or electrical stimulation, without altering basal [Ca^2+^]_i_, independently of SERCA [173]. Indeed, EGCG appears to be a powerful sensitizer that is highly selective toward potentiating RyR1 activity at lower concentrations, which does not directly affect SERCA [85]. On the other hand, higher EGCG concentrations (>10 μmol/L) produced an elevation of [Ca^2+^]_i_, which was correlated with the inhibition of SERCA activity [59]. Kargacin et al. [86] studied the effects of EGCG on SERCA2a turnover and Ca^2+^ transport in cardiac SR vesicles. EGCG increased the Ca^2+^-sensitivity of Ca^2+^ uptake into cardiac SR vesicles in a concentration-dependent manner, which was affected by the interaction between SERCA and PLB [86]. In another study, EGCG (>1 μmol/L) improved the contractility of cardiac muscle cells via increased Ca^2+^ loading into the SR, and facilitated the release of Ca^2+^ through RyR2 without affecting SERCA2 activity or L-type Ca^2+^ currents. Enhanced activity of RyR2 promoted increased amplitude of Ca^2+^ transients. Moreover, EGCG in nanomolar concentrations caused a positive inotropic effect [87]. Possible epigenetic regulation of SOCE inhibition by EGCG via suppression of ORAI1–STIM2 expression was reported. EGCG (10 μmol/L) downregulated the PTEN/mTOR pathway and decreased mitochondrial potential, as shown in murine and human Jurkat T cells [174].

Tannins are polyphenolic compounds found in a variety of plant-based foods and beverages, including tea. Low tannin concentrations (EC_50_ ≈ 0.3 μmol/L) were observed to stimulate Ca^2+^ uptake and ATP hydrolysis in cardiac SR, while higher concentrations (IC_50_ ≈ 3 μmol/L) inhibited SERCA2 activity in both cardiac and skeletal muscle SR. Therefore, the observed SERCA stimulation seems to result from removing the inhibition of SERCA by PLB, similarly to that observed by EA. The inhibitory effect of tannin was attributed to competitive inhibition of nucleotide binding [83]. Tannins were likewise reported to activate SERCA2 by acting on the inhibitory complex SERCA–PLB [101]. Recently, the protective effect of tannic acid (TA)—a representative of tannin—against vascular calcification in renal proximal tubular cells was reported. The TA-mediated mechanism of decreasing ER stress-induced cell death resulted partially from the inhibition of the SOCE pathway, reducing the intracellular Ca^2+^ overload [175]. Moreover, it has been suggested that TA might chelate available Ca^2+^ due to its galloyl groups [176] and, thus, may modulate Ca^2+^ dysregulation.

Caffeic acid (CA) is a phenolic phytonutrient classified as a hydroxycinnamic acid, which can be found in abundance in various plants, and possesses a wide range of beneficial properties. CA was shown to have positive impacts on vascular function and blood pressure by potentiating SERCA2a activity in the vascular smooth muscle cells. Binding of CA to SERCA was accompanied by the formation of strong hydrogen bonds. Specifically, CA restored the thapsigargin-induced rise in [Ca^2+^]_i_ in a concentration-dependent manner, and increased Ca^2+^ uptake into the SR [81]. Another study revealed that rosmarinic acid (RA)—a caffeic acid ester and polyphenol constituent of many culinary herbs—had cardioprotective effects against acute myocardial infarction, which was associated with increased gene expressions of both SERCA2 and RyR2 [99].

Bergamot (*Citrus bergamia*), a citrus fruit rich in the polyphenols neoeriocitrin, neohesperidin, naringin, bruteridin, and melitidin, exerts various pharmacologically beneficial properties—especially cardioprotective and antidiabetic effects [177]. Kang et al. [178] investigated the effect of bergamot on the intracellular Ca^2+^-regulating properties in endothelial cells. They concluded that bergamot increased the intracellular Ca^2+^ concentration via the release of Ca^2+^ from the intracellular Ca^2+^ stores, as well as through SOCE, suggesting protective effects on endothelial dysfunction [178].

## 5. Concluding Remarks

Ca^2+^-transport systems, including SERCAs, may represent interesting molecular targets for therapeutic interventions of pathophysiological states associated with Ca^2+^ dysregulation. The role of SERCA activation in the management of ER stress-related diseases has recently emerged as a promising pharmacological strategy. Dietary polyphenols, owing to their structural features (i.e., planar motif and/or numerous hydroxyl groups), are able to interact with SERCA and affect a wide range of intracellular events. Based on the reviewed information, polyphenolic substances may be beneficial in the management of chronic disease conditions associated with SERCA downregulation, such as cardiovascular diseases, diabetes, metabolic disorders, and neuropathological conditions.

To summarize the key mechanisms of polyphenols’ protective action with respect to impaired intracellular Ca^2+^ homeostasis, it is noteworthy to highlight the following:

(i) Upregulation of SERCA expression, specifically via AMPK/SIRT activation;
(ii) Increase in SERCA activity and stability;
(iii) Relieving SERCA2 from the SERCA–PLB complex;
(iv) Enhancing RyR1 and RyR2 activity/expression,
(v) Affecting Ca^2+^-dependent channels, such as L-type and T-type VGCCs, Ca^2+^-activated K^+^ channels, or SOCs.

Increasing SERCA activity/expression via specific SERCA activators may help to ameliorate ER stress while reducing cytosolic Ca^2+^ overload as well as relieving the load on the underlying adaptive Ca^2+^ regulatory pathways and, thus, contributing to the management of diseases associated with ER stress. Activation and upregulation of SERCA2 seems to be included within the core Ca^2+^-dependent mechanisms, whereby polyphenols manifest their cardioprotective and antidiabetic actions. However, additional studies (especially clinical trials) are required to address the detailed mechanisms of polyphenol-mediated action with respect to polyphenol–SERCA interactions and downstream Ca^2+^ signaling pathways to contribute in the management of various pathophysiological conditions.

## Figures and Tables

**Table 1:** The pathophysiological roles of SERCAs in human diseases.

SERCA Isoform	Tissue Distribution	Disease/Complication	SERCA Activity/Expression	Reference
SERCA1a	Adult fast-twitch skeletal muscle	Brody’s disease	↓/↓	[12,36]
SERCA1b	Fetal fast-twitch skeletal muscle	Myotonic dystrophy type 1	↓/↑	[12,37]
SERCA2a	Slow twitch skeletal muscle, cardiac muscle, smooth muscle cells	Heart failure Cardiac hypertrophy Diabetic cardiomyopathy Vascular complications Early type 2 diabetes	↓/↓ -/↓ ↓/↓ ↓/↓ -/↑	[12,13,38,39]
SERCA2b	All tissues (muscle and non-muscle cells)	Darier’s disease Type 1 and 2 diabetes Cancer Neurodegenerative diseases	↓/↓ ↓/↓ -/↓ ↓/↓↑	[12,36,40,41,42]
SERCA2c	Epithelial, mesenchymal, and hematopoietic cells; monocytes	Cardiomyopathy	-/↑	[28,36,43]
SERCA2d	Skeletal muscle	Myotonic dystrophy type 1	-/↓	[37]
SERCA3a-f	Non-muscle tissues	Type 2 diabetes Type 1 diabetes Cardiomyopathy Cancer	-/↓ -/SERCA3b↑ -/SERCA3f↑ -/↑↓	[39,41,43]

↓/↑ refers to decrease/increase in SERCA activity or down/upregulation of its expression.

**Table 2:** The most important mechanisms of action of natural polyphenols related to SERCA activation.

Compound (MW)	Structure	Mode of Action Related to SERCA	Study Model	Health Implications	Ref.
Baicalein; BAI (270.24)	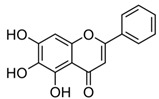	Upregulation of SERCA2 and RyR2, downregulation of CAMKII	Rats, H9C2 myocardial cells	Cardioprotection, alleviation of heart failure	[79,80]
Caffeic acid; CA (180.16)	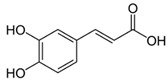	Activation of SERCA2a by direct binding	Wild-type mice	Improved vasoconstriction, lowered blood pressure	[81]
Ellagic acid; EA (302.197)	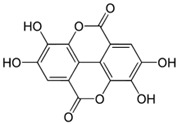	Activation of SERCA2 via removing PLB’s inhibition of SERCA	Myocardium from diabetic mice, cardiac SR vesicles	Amelioration of diastolic dysfunction, mediating cardiac contractile responses	[82,83,84]
(-)-Epigallo- catechin-3-gallate; EGCG (458.37)	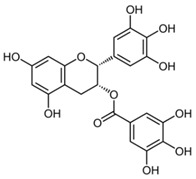	Enhancing RyR1 and RyR2 activity, and affecting SERCA via the interaction with PLB	Skeletal myotubes/myofibers, murine myocytes, cardiac SR vesicles	Improved contractility and muscle function, positive inotropic effects	[85,86,87]
6-Gingerol; GIN (294.38)	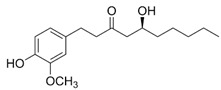	Direct SERCA1 and SERCA2 activation	Myocardium from diabetic mice, cardiac and skeletal SR vesicles, NG115-401L neuronal cells	Amelioration of diastolic dysfunction, mediating cardiac contractile responses	[82,84,88,89]
Luteolin; LUT (286.24)	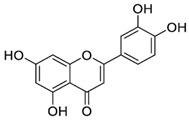	Improvement of SERCA2a expression, activity and stability, partially via SUMO1 and Sp1; increasing SERCA2a activity via suppression of p38 MAPK and activation of PI3K/Akt pathways	Cardiac HL-1 cells, C57BL/6J mice, cardiomyocytes, intact heart, ischemia–reperfusion rat model	Attenuation of myocardial Ischemia–reperfusion injury, improved systolic/diastolic function, amelioration of myocardium fibrosis and heart failure	[90,91,92,93,94]
Myricetin; MYR (318.23)	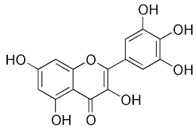	Upregulation of SERCA2b expression, partially via PDX1	INS-1 cells, isolated rat islets	Protection of beta cells from apoptosis, attenuation of type 2 DM	[95]
Resveratrol; RSV (228.24)	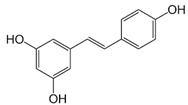	Upregulation of SERCA via SIRT1 activation	Mouse model of type 1 DM, Sprague–Dawley rats	Improvement of cardiac function in diabetes, prevention of cardiac hypertrophy	[96,97,98]
Rosmarinic acid; RA (360.32)	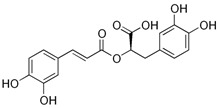	Upregulation of the expression of SERCA2 and RyR2	Sprague Dawley rats, isolated hearts	Cardioprotective effects against myocardial infarction and arrhythmia	[99]
Rutin; RUT (610.52)	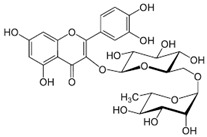	Stimulation of SERCA1 activity by direct binding	Skeletal SR vesicles	Potential significance in cardiovascular and skeletal muscle diseases	[100]
Tannic acid; TA (1701.19)	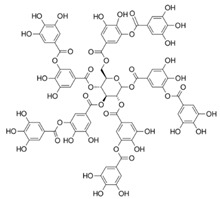	Activation of SERCA2 through relieving the inhibitory effect of PLB on SERCA	Cardiac SR vesicles	Pharmacological intervention in impaired cardiac contractility and function	[83,101]

## Data Availability

Data are contained within the review article.

## Abbreviations

AMPK, 5′ adenosine monophosphate-activated protein kinase; BAI, baicalein; Bcl-2, B-cell lymphoma 2; CA, caffeic acid; CaMKII, Ca^2+^/calmodulin-dependent protein kinase II; EA, ellagic acid; EGCG, (-)-epigallocatechin-3-gallate; ER, endoplasmic reticulum; GIN, 6-gingerol; IP3R, inositol-1,4,5-triphosphate receptor; IRE1, inositol-requiring enzyme 1; JNK, c-Jun NH2-terminal kinase; LUT, luteolin; mTOR, mammalian target of rapamycin; MAPK, mitogen-activated protein kinase; MCU, mitochondrial calcium uniporter; MYR, myricetin; NCX, Na^+^/Ca^2+^ exchanger; NF-κB, nuclear factor kappa B; ORAI, calcium release-activated calcium channel protein; PLB, phospholamban; PMCA, plasma membrane Ca^2+^-ATPase; PPAR, peroxisome proliferator-activated receptor; RA, rosmarinic acid; RSV, resveratrol; RUT, rutin; RyR, ryanodine receptor; SERCA, Ca^2+^-ATPase from sarco/endoplasmic reticulum; SIRT, NAD-dependent deacetylase sirtuin; SOCE, store-operated calcium entry; SOC, store-operated calcium channel; Sp1 transcription factor, specificity protein 1; STIM1, stromal interaction molecule 1; SUMO1, small ubiquitin-related modifier 1; TA, tannic acid; TRPC, transient receptor potential canonical channel; VGCC, voltage-gated calcium channel.
